# Autologous bone marrow expanded mesenchymal stem cells in patellar tendinopathy: protocol for a phase I/II, single-centre, randomized with active control PRP, double-blinded clinical trial

**DOI:** 10.1186/s13018-019-1477-2

**Published:** 2019-12-16

**Authors:** Gil Rodas, Robert Soler, Ramón Balius, Xavier Alomar, Xavier Peirau, Mercedes Alberca, Ana Sánchez, Javier García Sancho, Clementina Rodellar, Antonio Romero, Lorenzo Masci, Lluís Orozco, Nicola Maffulli

**Affiliations:** 1 Medical Department, Football Club Barcelona, C/Aristides Maillol, s/n 08028, Barcelona, Spain; 2 Medicine and Exercise Sport Unit, Hospital Clínic and Sand Joan de Deu, Barcelona University, C/Villarroel 170, 08036 Barcelona, Spain; 3 ITRT Institut Terapia Regenerativa Tissular Centro Médico Teknon, C/Vilana 12. 08022, Barcelona, Spain; 4 Consell Català del’Esport, Unitat d’Esporti Salut Av Països Catalans, 40-48, 08950 Esplugues, Spain; 5 Diagnóstico por la Imagen, Clínica Creu Blanca, Passeig de la Reina Elisenda de Montcada, 17, 08034 Barcelona, Spain; 6 Institut Nacional d’Educació Física de Catalunya (INEFC), Partida Caparrella s/n, 25192 Lleida, Lleida Spain; 7 Instituto de Biología y Genética Molecular (IBGM), Universidad Valladolid y CSIC Edificio IBGM, C/ Sanz y Forés, s/n, 47003 Valladolid, Spain; 8 Red TerCel de Terapia Celular, Instituto de Salud Carlos III, Hospital Clínico Universitario Virgen de la Arrixaca. Servicio de Hematología. Edf. General, Ctra. Madrid-Cartagena s/n, 30120 Murcia, Spain; 9 LAGENBIO, Facultad de Veterinaria, Lab. Genética Bioquímica. Facultad de Veterinaria, Universidad de Zaragoza, C/Miguel Servet 177, 50013 Zaragoza, Spain; 10 Institute of Sport Exercise and Health, London, UK; 11 Department of Musculoskeletal Disorders, University of Salerno School of Medicine, Surgery and Dentistry, Salerno, Italy; 12 Centre for Sports and Exercise Medicine, Queen Mary University of London, London, UK; 13 School of Pharmacy and Bioengineering, Keele University School of Medicine, Stoke on Trent, UK

**Keywords:** Tendinopathy; Mesenchymal stem cells; Platelet-rich plasma

## Abstract

**Introduction:**

Patellar tendon overuse injuries are common in athletes. Imaging may show a change in tissue structure with tendon thickening and disruption of the intratendinous substance. We wish to test the hypothesis that both autologous bone marrow expanded mesenchymal stem cells and autologous leukocyte-poor platelet-rich plasma (LP-PRP) implanted into the area of the disrupted tendinopathic patellar tendon will restore function, but tendon regeneration tissue will only be observed in the subjects treated with autologous bone marrow expanded mesenchymal stem cells.

**Methods and analysis:**

This is a single-centre, pilot phase I/II, double-blinded clinical trial with randomisation with active control. Twenty patients with a diagnosis of patellar tendinopathy with imaging changes (tendon thickening and disruption of the intratendinous substance at the proximal portion of the patellar tendon) will be randomised in a 1:1 ratio to receive a local injection of either bone-marrow autologous mesenchymal stem cells (MSC), isolated and cultured under GMP at T*he Institute of Biology and Molecular Genetics* (IBGM) (Spain) or P-PRP. The study will have two aims: first, to ascertain whether a clinically relevant improvement after 3, 6 and 12 months according to the visual analogue scale (VAS), Victorian Institute of Sport Assessment for patellar tendons (VISA-P) and dynamometry scales (DYN) will be achieved; and second, to ascertain whether the proposed intervention will restore tendon structure as determined by ultrasonography (US), Doppler ultrasonography (DUS), and innovative MRI and ultrasound techniques: Magnetic Resonance T2 FAT SAT (UTE, Ultrashort Echo TE) sequence and Ultrasound Tissue Characterization (UTC). Patients who are randomised to the P-PRP treatment group but do not achieve a satisfactory primary endpoint after 6 months will be offered treatment with MSC.

**Trial registration:**

NCT03454737.

## Background

Overload patellar tendon injuries are common in elite athletes (7 to 45%) and activities where repetitive jumping is required [1–4]. Pain may become recurrent and refractory to any conservative management, leading to a sports-related or occupational disability [5]. The pathogenesis of patellar tendinopathy is still elusive, but a failed healing response remains at its basis [6, 7]. At the time of surgery, typically no inflammatory cells or inflammatory mediators are detected in the tendon tissue, suggesting that tendinopathy is not the result of typical inflammation [8, 9]. Poor vascularization is often reported as a factor that contributes to tendinopathy, but tendinopathy is often associated with neovascularization and increased blood-flow through the tendon, which might be a restorative and beneficial response [10]. Although the source of pain has not been defined [11], in some studies small substance P-positive nerve fibres are closely related to the neovascularization process and may transmit pain perception [6, 12–14].

Other pathological changes related to patellar tendinopathy include (a) intra-tendinous calcification; (b)accumulation of adipose cells; (c) disruption and disorganisation of collagen fibres; (d) conversion of type I collagen to type III collagen; (e) a reduction in number of fibroblasts; (f) changes in tenocyte morphology; and (g) an increase in proteoglycan and glycosaminoglycan content [6].

Although the accepted standard treatment for patellar tendinopathy is a graded rehabilitation programme, not all patients respond to exercise therapy [15]. Medical or surgical management may provide improvement in symptoms but does not result in regeneration of normal tendon tissue [16]. Recently, MSC have been shown to promote tendon healing in an animal model of acute tendon injury [17, 18]. In addition, these cells may control inflammation by influencing differentiation, migration or apoptosis in different immune cells [19–25].

MSC have the following distinctive features: (a) ability to differentiate into osteoblasts, adipocytes and chondrocytes in vitro; (b) ability to adhere to plastic during culture; (c) more than 90% of the cells show a positive immunophenotype for CD73, CD105, CD90 and CD166 antigens; less than 10% of the cells show a positive immunophenotype for CD45, CD14, CD34, CD31 and HLA-DR antigens [26]. MSCs are found in several organs and tissues, where they may be harvested. Nevertheless, bone marrow is the optimal and most easily accessible source of MSC. Once obtained, these cells can be isolated and grown *ex vivo* under pharmaceutical standards and used in human therapies known as an ‘advanced therapy medicine’ [27]. Our research group has used MSC successfully in several osteoarticular injuries [28–36]. Given the immunomodulatory and anti-inflammatory abilities of MSC, harvesting and grafting these cells into an area affected by patellar tendinopathy are feasible options that may provide satisfactory results.

The use of autologous leukocyte-poor platelet-rich plasma (LP-PRP) in tendon injuries is well established [37], including patellar tendon injuries, at times showing good clinical response [38, 39]^.^ Our research group has also successfully used this approach in various musculoskeletal conditions in several areas of the human body [40, 41]. A recent systematic review and meta-analysis [42] demonstrated improved outcomes with the use of leukocyte-rich-PRP (LR-PRP) compared to LP-PRP.

We wish to test the hypothesis that an analgesic effect will be achieved using either treatment, but significant regeneration of tendon tissue will only be achieved in the cell therapy group. We will assess clinical results by clinical examination, visual analogue scale (VAS), the validated Spanish version of VISA-P^43^ and manual dynamometry. We will evaluate tissue regeneration using several imaging techniques. The use of MRI with a UTE sequence in the sagittal plane makes it possible to determine tendon tissue features at very low T2 values in all regions, including the ‘gap’ and healthy tendon tissue. In addition, UTE sequences allow to visualize and delineate defects that are not detected by conventional imaging.

UTC is an innovative imaging technique for assessing tendon injuries. Using a conventional ultrasound device, 600 axial sections of the tendon are performed at 0.2 mm intervals and compounded to form a 3D image. Using this technique, collagen fibre alignment may be quantified into four different types: (a) normal aligned fibrillar structure; (b) slightly misaligned fibrillar structure; (c) disorganised fibrillar structure; (d) complete disintegration of tendon tissue [43]. Ultrasonography is the most accessible imaging technique used to confirm the clinical diagnosis and determine the exact location of the lesion. Ultrasonography also helps calculate tendon size, defines eco-texture, allows measurement of any tendon gaps in several planes and assesses the degree of tendon vascularization. In addition, ultrasonography allows to dynamically monitor tendon function [44].

Should the results be better for the cell therapy group, the patients in the active control group (who were injected with LP-PRP) who have not achieved similar benefits to those of patients in the MSC group after 6 months will be offered cell therapy. We believe that this will increase the feasibility of the clinical trial.

### Characteristics of the MSC intervention

MSCs exhibit the typical stem cell features defined by the International Society of Cellular Therapy [23]. MSC are an advanced therapy drug registered with the *Agencia Española de Medicamentos y Productos Sanitarios* (AEMPS) with IND number 10–134. They are bone marrow MSC, processed according to the GMP practices at the IBGM and re-suspended in an isotonic medium composed of Ringer lactate solution, 0.2% human serum albumin and 5mM glucose. The product is supplied by the laboratory after a 23-day processing period. Cell viability is ≥ 90%, and negative microbiological results are obtained for Gram and Mycoplasma microorganisms. Endotoxin levels are below 0.5 IU/mL. The product is stable for 8 h at 4–12 °C. The product is supplied in two 5 mL syringes, one containing 10 × 10^6^ MSC suspended in a 2 mL solution, and the other containing 10 × 10^6^ MSC suspended in a 4 mL solution. The total dose of cells supplied is 20 × 10^6^ ± 2 × 10^6^ MSC.

### General characteristics of P-PRP intervention

LP-PRP is obtained under strict aseptic conditions. Blood components are fractioned by centrifugation. In a surgical environment, peripheral venous blood is collected into four 9 mL citrate tubes. The anticoagulated blood is centrifuged at 1,200 rpm for 8 min (460*g*). Six milliliters of LP-PRP are obtained from a 36-mL sample of autologous peripheral venous blood. The LP-PRP suspension contains a two-fold increase in the number of platelets compared to peripheral blood and a very low number of leukocytes (average basal peripheral blood 228 × 10^3^ platelets/mm^3^, range 165–329 × 10^3^; LP-PRP: 563 × 10^3^ platelets/mm^3^; range 407–801 × 10^3^). Before application, plasma coagulation is activated by adding 5% CaCl2 [38, 45].

## Methods/design

### Main objectives

1. To assess the clinical efficacy of MSC injection and compare the results with those obtained in the LP-PRP group. Outcomes will be assessed using subjective patient reports, VAS and VISA-P scales, and strength determination of the extensor muscle group using manual dynamometry (DYN).
2. To confirm changes in the architecture for regeneration of the patellar tendon gap following peritendinous and intratendinous injection of MSC and compare these results with those obtained in the LP-PRP group. Assessment will be performed using UTE-MRI, UTC and ultrasonography.

### Secondary objectives

To assess the viability and safety of the use of medical devices MSC and LP-PRP when injected into the patellar tendon; prove that all procedures described in the present protocol are feasible; and register potential adverse events related to either treatment, as well as any other adverse events that occur during the clinical trial, whether related to the trial or not.

### Trial design

#### Study population

**Fig. 1:**
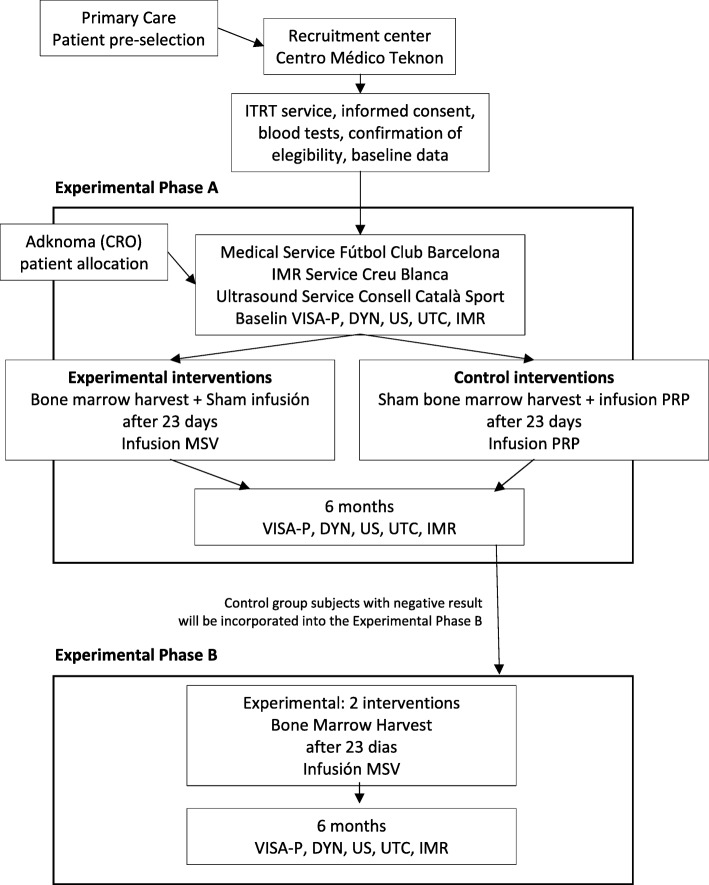
Study flow chart

Twenty male recreational athletes with a patellar tendinopathy who meet the inclusion and exclusion criteria will be recruited and will be followed for 1 year (Fig. 1).

Inclusion criteria:

1. Male participants aged between 18 and 48
2. Pain and tenderness at the area of attachment of the patellar tendon on the lower pole of the patella [46] for more than 4 months, with no clinically relevant response after conservative treatments
3. US confirming disruption ≥ 3 mm of the fibrillar structure in the proximal portion of the patellar tendon, tendon thickening and a hypoechoic injury MRI of the patellar tendon in T2 FAT SAT sequence (fat saturation) showing an increase of ≥ 3 mm in longitudinal diameter at the proximal insertion
4. Informed signed written consent by the participant
5. The participant is able to understand the nature of the study.

Exclusion criteria:

1. Participant younger than 18 (or legally dependent) or over 48 years of age
2. MRI with a grade III–IV osteochondral lesion in any compartment of the knee
3. Injury to the anterior or posterior cruciate ligament
4. Local injection with corticosteroid during the previous 12 months
5. Local injection with PRP during the previous 6 months
6. Evidence of local or systemic infection
7. Patients presenting with positive serology to HIV 1 or 2, hepatitis B (HBsAg, HBcAc), hepatitis C (anti-HCV-Ab) and syphilis
8. Congenital or acquired conditions involving malformation and/or significant deformation of the knee that hampers the administration of the planned intervention and evaluation of the results
9. Body mass index (BMI) greater than 30. 5 (obesity grade II)
10. Active neoplastic disease
11. Immunosuppression
12. Simultaneous participation in another clinical trial or treatment with another research product during the 30 days prior to inclusion in the study
13. Other pathologies or circumstances that may compromise participation in the study according to medical criteria.

Patients will be randomized by the Clinical Research Organization (CRO) (Adknoma) using SAS statistical software (version 9. 4) into either the MSC group or PRP group in a 1: 1 ratio. After randomization, either MSC or LP-PRP will be injected intralesionally and around the affected area of the patellar tendon. To keep patients and evaluating physicians blind to treatment, sham procedures will be performed in both groups (i.e. the MSC group will have blood extracted and will have their tendon injected with normal saline at the first stage of treatment, and the LP-PRP group will undergo a sham bone marrow harvesting at the first stage of treatment). The treating physicians will not be blind to the treatment administered.

### Experimental phase A

#### MSC treatment group

Ten patients will be treated with saline solution (sham) and with 20 × 10E^6^ MSC after 23 days.

##### Stage one

1. Under sterile conditions, a total of 4 citrate tubes (9 mL per tube; total volume 36 mL) of venous blood are withdrawn from the antecubital vein of the non-dominant arm as a sham procedure of P-PRP preparation.
2. With the patient prone, and after sedation induced by midazolam and propofol, local anaesthetic injections (lidocaine 1%; 10 mL) are administered in both the posterior superior iliac crests and are penetrated using an 11 gauge trocar. Using sequential aspirations of 1–2 mL each, 100 mL of bone marrow is collected in a heparinized container to be sent to IBGM for isolation and culture of MSC.
3. The patient is then positioned supine. Under ultrasonography guidance, 1 mL of normosaline solution is injected using a 22 gauge needle in the tendinous and 1 mL in the paratendinous medial and lateral areas of the patellar tendon. The participant is then transferred to the postoperative recovery room, where they remain for 1 h before discharge.

##### Stage two (after 23 days)

1. Under sterile conditions, a total of 4 citrate tubes (9 mL per tube; total volume 36 mL) of venous blood are withdrawn from the antecubital vein of the non-dominant arm as a sham procedure of P-PRP preparation.
2. With the patient supine under sedation with midazolam and propofol, under ultrasonography guidance, a 2-mL solution containing 10 × 10^6^ MSV is injected into the tendon; a 2-mL solution containing 5 × 10^6^ MSC is injected into the medial peritendinous area, and a 2-mL solution containing 5 × 10^6^ MSC is injected into the lateral peritendinous area.

#### LP-PRP control group

Ten patients will be treated with two doses of LP-PRP, each containing 6 mL of solution-PRP. The second dose will be injected 23 days after the first one.

##### Stage one

1. Under sterile conditions, a total of 4 citrate tubes (9 mL per tube; total volume 36 mL) of venous blood are withdrawn from the antecubital vein of the non-dominant arm for P-PRP preparation. One to two supplementary tubes of peripheral venous blood are withdrawn for preservation in a serum bank for security reasons. LP-PRP is prepared according to ITRT’s PRP protocol.
2. With the patient prone, and after sedation induced by midazolam and propofol, 10 mL of lidocaine 1% is administered in the posterior superior iliac crests, and an 11-gauge trocar is inserted in the subcutaneous tissue as part of the sham procedure for obtaining MSC.
3. With the patient supine, and sedation induced by midazolam and propofol, using ultrasound guidance, 2 mL of LP-PRP are injected intratendinously, 2 mLof LP-PRP are injected into the medial peritendinous area, and 2 mL of LP-PRP are injected in the lateral peritendinous zone adjacent to the area of tendinopathy.

##### Second stage (after 23 days)

1. The procedure for obtaining blood and LP-PRP preparation is performed again under sterile conditions.
2. With the patient supine, and sedation induced by midazolam and propofol, using ultrasound guidance, 2 mL of LP-PRP are injected intratendinously, 2 mL of LP-PRP are injected into the medial peritendinous area, and 2 mL of LP-PRP are injected in the lateral peritendinous zone adjacent to the area of tendinopathy.

### Experimental phase B

Results will be evaluated 6 months after the treatment. If the results support the hypothesis that a higher efficacy is obtained in the MSC group, the cell therapy trial will be offered to subjects from the LP-PRP group who show no evidence of clinically relevant improvement: if they wish, this group will be treated with MSC. This will be ‘experimental phase B’, which will be evaluated with the same clinical and imaging assessment protocol as described above.

### Post-treatment procedures (rehabilitation program)

The participants will be discharged 1 h after injection of the cell suspension or LP-PRP solution and advised to rest for 48 h. Analgesia (ibuprofen 600 mg, every 8 h, and omeprazole 20 mg once daily) are prescribed for the participant to self-administer if needed.

After treatment, a specific rehabilitation protocol will be followed, applying appropriate progressive load to the affected limb [6]. Table 1 shows the rehabilitation program over 3 months. For greater details, please refer to Malliaras et al 2015 [15] and Mascaro et al. 2018 [47].

**Table 1:** Rehabilitation program over 3 months

Rehabilitation program	Day 0–3	4–5 days	2–3 weeks	4–8 weeks	3 months
RICE					
Isometric squat against wall		5 reps × 30–40 s (3’ rest). Progress from 45° to 90° knee flex)			
Cycling (alternate days)			15–30 min	15 min	
Isometric leg extension 30°			8 reps × 45'' (1’ rest)		
Elliptical training (alternate days)				15 min	
Slow dynamic knee extension (Con/Ecc)				4'' Con/4'' Ecc, 30'' rest. Increase load from 2 to 12 kg. Progress from bilateral to unilateral	
Numeric rate score (NRS) allowed				< 4/10, if > 4/10 stop progression load for 2–3 days	
Running					Progressively
Plyometrics					Progressive jumps landing to plyometrics. Every 3 days
Fast dynamic exercise (Con/Ecc)					3 × 6–8 reps, 1–3' rest

After the 3 months, and after evaluating each patient by physical examination, US and MRI, the athletes will be progressively allowed to gradually return to their sport.

The follow-up consists of clinical examination visits and image monitoring by ultrasonography and by UTC, MRI-UTE, as specified in the protocol schedule.

### Concomitant medications

Any concomitant medications administered from the day of the initial treatment until the end of the trial will be recorded. No fluoroquinolone-based antibiotic drugs will be administered during the entire study period [48, 49]. If they must be administered, the subject will be excluded from the clinical trial and will continue therapy using standard treatments. Corticosteroids or anti-inflammatory therapy should not be administered. If they must be administered, the patient will not be excluded from the trial, but the administration will be recorded in the clinical records. If necessary, treatment with ibuprofen 600 mg will be allowed.

### Response evaluation

Subsequent assessments following the treatment include AE review, VAS (pain), VISA-P Questionnaire, ultrasound imaging (conventional ultrasound, UTC and MRI), dynamometry manual test and clinical assessment (Table 2 for phase A, and Table 3 for phase B). These will take place at 1, 2, 3, 6, 8 and 10 weeks and 3, 6, 12 months post-treatment.

**Table 2:** Outcome assessments experimental phase A

Study period															
	Enrollment				Treatment		Follow-up								
Timepoints					0	23 days	1 week	2 weeks	3 weeks	6 weeks	8 weeks	10 weeks	3 months	6 months	12 months
Inf. sheet	X														
Incl/excl Criteria		X													
IC		X													
Preop + serology			X												
Allocation				X											
ClinicalHistory	X	X													
Physical exam	X	X					X	X	X	X	X	X	X	X	X
VAS		X					X	X	X	X	X	X	X	X	X
VISA-P		X					X	X	X	X	X	X	X	X	X
blood test													X		
Group MSV BM					X										
Group MSV saline sol.					X										
Group MSV MSV						X									
Group PRP BM sham					X										
Group PRP PRP					X	X									
Bloodbank					X										
MRI		X											X*		X
Ultrasound		X					X	X	X	X	X	X	X	X	X
UTC			X						X		X		X	X	X
DYN			X						X		X		X	X	X
AE					X	X	X	X	X	X	X	X	X	X	X
Medication					X	X	X	X	X	X	X	X	X	X	X

*Elig.* eligibility, *IC* informed consent; *VAS* visual analogue scale; *VISA-P* Victorian Institute of Sport Assessment (Patellar); *BM* bone marrow harvest; MSC Infusion of MSC; *MRI* magnetic Rresonance; *UTC* ultrasound tissue characterisation; *DYN* dynamometry, *PreOp* preoperative study, *AE* Adverse Events.

X* if the *VAS* and *VISA-P* scales show a significant improvement, and *MRI*, *US*, and UTC provide evidence of healing, the result will be considered positive and the patient will be followed up with confirmation controls at 6 and 12 months. For safety reasons, according to AEMPS criteria, a control test will be performed after the trial, within a period of 2 years after treatment.

If the 6-month evaluation shows negative results, patients may be transferred to the open experimental phase B.

**Table 3:** Outcome assessments experimental phase B

Study period															
	Enrollment			Treatment		Follow-up									
Timepoints				0	23 days	1 weeks	2 weeks	3 weeks	6 weeks	8 weeks	10 weeks	3 months	18 weeks	6 months	12 months
IC		X													
Preop + serology			X												
Physicalexam	X	X				X	X	X	X	X	X	X	X	X	X
VAS		X				X	X	X	X	X	X	X	X	X	X
VISA-P		X				X	X	X	X	X	X	X	X	X	X
blood test												X			
BM				X											
MSV					X										
Bloodbank				X											
MRI		X										X*	X	X	X
Ultrasound		X				X	X	X	X	X	X	X	X	X	X
UTC			X					X		X		X	X	X	X
DYN			X					X		X		X	X	X	X
AE				X	X	X	X	X	X	X	X	X	X	X	X
Medication				X	X	X	X	X	X	X	X	X	X	X	X

*VAS* visual analog scale; *VISA-P* Victorian Institute of Sport Assessment (Patellar); *BM* bone marrow harvest; *MSV* injection of *MSV*; *MRI* magnetic resonance imaging; *UTC* ultrasound tissue characterization; *DYN* dynamometry, *PreOp* preoperative study, *AE* adverse Events.

X* this MRI test will establish the status of the healing process at 3 months.

### Adverse events

Possible adverse events occurring during the trial period (regardless of whether they are related to the procedure) will be recorded and tabulated. In the event of a serious adverse effect, a notification will be sent to the Spanish agency for Medicines and Healthcare Products (AEMPS) through the Clinical Research Organization (CRO), according to established procedures and terms.

### Pain and function

Pain will be recorded on the VAS, and function will be evaluated by measuring extension forces in the knee using a manual dynamometer.

The pain and functional evaluation will be performed using the VISA-P score, which specifically evaluates pain and function in patellar tendinopathy. The scale ranges from 0 to 100, where a value of 100 indicates the absence of pain at rest, when walking, when climbing up or downstairs or during exercise [50]. VISA-P score values for patients with chronic patellar tendinopathy usually range from 30 to 50. Physical activity may be undertaken again if values are above 60. Patients with score values over 70 are considered fit for competition, although demands are higher for professional athletes. The minimum significant difference has been set at 13 points.

The following VISA-P criteria are used to assess improvement in this trial [51]:

- Desired: ≥ 60/100 (after 3 months)
- Very satisfactory: ≥ 70/100 (after 6 months)
- Ideal: ≥ 80/100 (after 12 months)

### Ultrasonography

Ultrasound scanning will be performed with an Aplio 500 (TUS-500 5.0 Platinum Series, manufactured by Canon Medical Systems Corporation in Nasu, Japan) using a high-frequency linear array probe (PLT 1005BT), 5.0–14 MHz frequency range. The most commonly used 2D frequency will be differential harmonic of 14 MHz (diff THI 14 MHz). The depth used will be 5 cm, with a single focus at 1.8 cm and a dynamic range of 65 dB

The patellar tendon will be examined longitudinally and transversely using greyscale and colour measures, exerting the least possible pressure with the probe. Images will be recorded in static and dynamic formats.

Therapeutic results will be regarded as positive if the following findings will be evidenced: improvement in tendon echotexture, gap reduction and reduced neovascularization.

A change in thickness will also be considered, but to a lesser extent, as the significance of thickness change in terms of success of any intervention programme is unknown [44].

The following will be determined:

1. Lesion location (medial, central, lateral) and (anterior, central, posterior).
2. Tendon thickness 5 mm distal to the inferior pole of the patella.
3. Eco-texture (using a 0–3 scale).
4. Longitudinal and axial “gap” measures; area value.
5. Vascularization degree I–V

### UTC

Tendon eco-structure will be evaluated according to four subtypes I, II, II and IV in cross-section of region of interest (ROI): percentage of each subtype will be calculated. The distance (mm) from the lower patellar pole in the anteroposterior section and cross-section will also be measured (based on percentage points).

Subtype I is regarded as ideal, corresponding to a perfectly aligned fibrillar structure; Subtype IV is regarded as the most sub-optimal, corresponding to the absence of fibrillar structure. To establish a healing standard, the 4 aforementioned subtypes will be assessed, and changes will be evaluated by calculating the relative increase in percentage points of subtypes I and II (indicative of organised tissue), along with the relative decrease in percentage points of subtypes III and IV (indicative of disorganised tissue).

### MRI

Regarding MR, we shall use a 3D sequence using a Toshiba TITAN 3T MRI (Canon Medical Systems, Corporation in Nasu, Japan), with a phase 3D MPV Voxel mVox T2 weight, isotropic Voxel (0.6 mm, 90.6 mm and 90.6 mm).

With a T2 FAT SAT sequence (fat saturation) in the sagittal plane, the dimensions of the intra-tendinous changes will be measured using several signal characteristics: hypo-signal, homogeneous signal, heterogeneous signal, isointense signal, and normal signal. Proximal, medial and distal areas of altered signal will be measured in millimetres. Hoffa fat oedema and patellar bone oedema will also be evaluated. An FFE (Fast FE) 3D sequence will be used in the coronal plane to measure the gap and the gap in cross-section diameter in millimetres. The presence of calcification will also be assessed. UTE (Ultrashort Echo TE) sequences will be obtained in the sagittal plane, and T2 mapping will be acquired in regions of Interest (ROI), and will be established for the areas of altered signal, in the medial and lateral areas, and in healthy tendon.

The intra-articular appearance of the knee joint will also be assessed, with quantification of the degree of arthrosis (I–IV) of each knee compartment.

### Criteria for non-response to treatment

A lack of response to treatment will be recorded if no clinically relevant improvement or tendon repair changes occur after a 6-month period. Clinical changes will be assessed using a combination of physical examination, VAS score, VISA-P questionnaire and DYN. Tendon repair will be assessed using ultrasonography, UTC and MRI. A partial response will be considered if there is evidence of either clinical improvement or tendon repair.

### Patient and public involvement

Patients and the public were not directly involved with the development of the current protocol. The primary research question is the efficacy of the use of culture-expanded MSCs versus LP-PRP on the treatment of patellar tendinopathy. This will enable the development of a larger RCT that will involve oversight from the patient and public representatives. Patient recruitment is described above, and the results will be disseminated to the participants directly in the doctor's office at the end of the study.

### Statistical analysis

Statistical analysis will be performed using version 21. 0 of the Statistical Package for Social Sciences (SPSS Inc, Chicago, IL). We will evaluate: (i) adverse events (AE) and serious adverse events (SAE) showing relationships, causality and severity will be provided; (ii) frequency and percentage of AE and SAE; (iii) laboratory results including hematologic and biochemistry values will be collated: leukocytes levels, absolute neutrophils count, platelet count, creatinine levels, AST (SGOT)/ALT (SGTP) levels, etc. A list of laboratory parameters will be obtained for each patient and visit and will include reference range values. Values will be summarized in a basal and post-basal visit analysis will include the following values: (i) valid ‘n’; (ii) mean; (iii) standard deviation; (iv) median; (v) minimum and maximum.

The frequency and percentage values of patients showing evidence of tendon regeneration 6 months after treatment with MSC or P-PRP will be provided.

The following methods will be used to describe and analyse the results obtained:

1. Descriptive statistics in numerical variables: number of valid cases; number of non-available values; mean value; standard deviation; 95% confidence interval for the mean value; median, minimum and maximum values. In discrete and categorical variables, the following values will be provided: frequency; percentage value for the total number of valid cases; percentage value for the total number of non-available values.
2. Inferential statistics tests for comparing groups and time points: ANOVA with repeated measures (or ANCOVA) if covariables with a significant interaction are detected with their corresponding contrast values (Scheffe test), or non-parametric tests if the assumption of normality is rejected (Mann-Whitney *U* test for comparison between groups and Wilcoxon test for comparison between measurements). The significance level for all statistical analyses will be 0. 05.

Efficiency sample (per protocol (PP) population) includes all patients whose tendon lesion has been treated with an MSC or LP-PRP suspension. These patients must have had no major violations of the protocol, and at least one assessment of efficacy after treatment.

‘Full analysis set’ (FAS) (intention to treat analysis) includes all patients in the study, whether they finish the study or comply with the protocol.

The safety population will be used in the analysis of the primary endpoint and of all other safety parameters defined.

A list of all demographic and baseline data will be provided for each patient. All lists will be provided for FAS and PP populations.

## Discussion

Patellar tendinopathy mainly occurs in young adults who practise sports, either professionally or as amateurs, which involve repeated running, hopping and jumping [52], with typical clinical and imaging diagnostics features [53].

To minimise uncontrolled factors that could affect the results, this study only includes male patients, as the viscoelastic properties of skeletal muscle and tendon are driven by hormone expression [54]. Disorders of insulin and thyroid hormones metabolism are related to tendinopathy both in vitro and in vivo [55], and sex hormones may promote changes in the structure of tendons, thus influencing their response to physical exercise [56]. If we obtain positive results in this study, a new study will be performed on female subjects only.

Both the cellular and applied LP-PRP treatments are based on safety and efficacy criteria obtained in animal studies and in clinical trials in which these treatments were applied successfully to treat other musculoskeletal pathologies [17, 18, 22, 29, 31–33, 41]. Future studies should try to establish the optimal cellular dose or the appropriateness of multiple doses. The composition and volume of the cell suspension medium should also be noted, as these variables can influence results.

Applying the guidelines of the Helsinki declaration of the World Medical Association, we have avoided using a true placebo in the control group, and plan to use an active control LP-PRP group. We hypothesise that this control treatment is very unlikely to exert the significant regenerative effect that we would attribute to MSC. However, this hypothesis must be confirmed, as no previous clinical trials have analysed the therapeutic effect of expanded BM-MSCs in patellar tendinopathy. The present study has several trengths. This is the first study to assess the effect of injecting autologous mesenchymal cells into the pathological zone of refractory proximal patellar tendinopathy in athletes. The study will monitor clinical scores, strength and imaging findings including 2D US, UTE MRI and ultrasound tissue characterization (UTC). The addition of UTE MRI and UTC will enable more precise measurement of structural tendon changes than 2D US. The control group will be offered mesenchymal cell therapy if the results of the control group prove ineffective. We are fully aware of the limitations of the present study. For example, the control group will receive active LP-PRP treatment rather than a true placebo, limiting the value of a control group. However, it will be easier to recruit athletes in a study with a control treatment that is perceived to be effective in an athletic cohort. The sample size is small, limiting the power of this study. Finally, this study fails to establish an optimal cell dose, which should be examined in future studies.

It is important to plan a second open experimental phase, where patients treated with LP-PRP who have not improved will be offered cell treatment. This approach, in addition to providing subjects included in the control group with a potentially more effective treatment, should also help determine the scope of the therapeutic effect of the MSC on LP-PRP, establishing a comparative cost-effective index.

This clinical trial also aims to advance current knowledge about the pathology of interest, and the hypothetical regenerative effect of incorporating innovative diagnostic systems and evaluations of this type of lesions. Both UTE magnetic resonance (Ultrashort Echo TE) and UTC are innovative non-invasive techniques that allow us to quantify regenerative effects using imaging.

## Trial Status

- Protocol version: V2. 05-July 2017
- Recruitment starts December 2017.
- Total subjects recruited on 28-10-2018: 20

## Data Availability

Not applicable.

## Abbreviations

AE (AA): Adverse event
AEMPS: *Agencia Española de Medicamentos y Productos Sanitarios* (Spanish Agency for Medicines and Healthcare Products)
CEIM: Ethics Committee for ClinicalResearch with Drugs
CRO: Clinical Research Organization
DYN: Dynamometry
FAS: Full analysis set
FFE: FAST FE Sequence (Ultrafast Gradient Echo Sequence)
GMP: Good Manufacturing Practices
IBGM: *Instituto de Biología Genética Molecular* (Institutefor Molecular Biology and Genetics)
ITRT: *Institut de TerapiaRegenerativaTissular* (Institute for Tissue Regeneration Therapy)
LP-PRP: Leukocyte-poor platelet-rich plasma
MO: Bone marrow
MRI: Magnetic resonance imaging
MSC: Mesenchymal stem cells
MSV: Mesenchymal stem cells from valladolid
PEI (IMP): Investigational medicine product
PP: Per protocol analysis
PRP: Platelet-rich plasma
ROI: Region of interest
SAE (AAG): Serious adverse event
T2: T2 sequence
UTC: Ultrasound tissue characterisation
UTE: Ultrashort echo time sequence
VAS: Visual analogue scale for pain
VISA: ‘Victorian Institute of Sport Assessment’ scale
VISA-P: ‘Victorian Institute of Sport Assessment (patellar)’ scale
